# Study of drug resistance-associated genetic mutations, and phylo-genetic analysis of HCV in the Province of Sindh, Pakistan

**DOI:** 10.1038/s41598-023-39339-4

**Published:** 2023-07-27

**Authors:** Sirmast Faiz, Muhammad Irfan, Saba Farooq, Ishtiaq Ahmad Khan, Hana’a Iqbal, Atia-tul Wahab, Muhammad Shakeel, Peng Gong, Thomas Iftner, M. Iqbal Choudhary

**Affiliations:** 1 Dr. Panjwani Centre for Molecular Medicine and Drug Research, International Center for Chemical and Biological Sciences, National Institute of Virology, University of Karachi, Karachi, 75270 Pakistan; 2 Dr. Panjwani Centre for Molecular Medicine and Drug Research, International Center for Chemical and Biological Sciences, Jamil-ur-Rahman Center for Genome Research, University of Karachi, Karachi, 75270 Pakistan; 3 Dr. Panjwani Centre for Molecular Medicine and Drug Research, International Center for Chemical and Biological Sciences, University of Karachi, Karachi, 75270 Pakistan; 4 Dr. Panjwani Centre for Molecular Medicine and Drug Research, International Center for Chemical and Biological Sciences, H.E.J. Research Institute of Chemistry, University of Karachi, Karachi, 75270 Pakistan; 5 Wuhan Institute of Virology, Chinese Academy of Sciences, No.44 Xiao Hong Shan, Wuhan, 430071 Hubei China; 6 Institute for Medical Virology and Epidemiology of Viral Diseases, University Hospital and Medical Faculty, Eberhard Karls University, Tuebingen, Germany

**Keywords:** Microbiology, Molecular biology, Diseases, Molecular medicine

## Abstract

Current management of HCV infection is based on Direct-Acting Antiviral Drugs (DAAs). However, resistance-associated mutations, especially in the NS3 and NS5B regions are gradually decreasing the efficacy of DAAs. The aim of the current study was to identify such mutations in the NS3, and NS5B genes in DAAs treatment-naïve Pakistani chronic HCV 3a patients. Peripheral blood samples were collected from 233 chronic HCV 3a patients at different tertiary care hospitals in Karachi, Pakistan, between August 2020 to September 2021. PCR-amplified target regions of the NS3/NS5B gene were subjected to Sanger sequencing to identify resistance-associated mutations. Phylogenetic analysis of the identified amino acid sequences was performed using HCV3a sequences of the global population in the virus pathogen resource (VIPR) database. Sequence analysis identified five amino acid mutations, Leu36Pro, Gln41His, Gln80Lys/Arg, Ala156Tyr, and Gln168Arg in the NS3 region, and two mutations Leu159Phe and Cys316Arg in the NS5B region. Phylogenetic analysis revealed a high genetic diversity in the studied isolates. Overall, the prevalence of resistance-associated substitutions was almost similar to other geographic regions worldwide. This data could be helpful in selecting the most effective treatment regimen for HCV chronically infected people in Pakistan.

## Introduction

The Hepatitis C virus (HCV) is a major cause of chronic hepatitis, hepatocellular carcinoma, and liver cirrhosis^1^. According to the WHO, 58 million people live with chronic infections of HCV, and the associated death toll is 0.29 million people annually^2^.

HCV is a highly diverse virus, including 8 genotypes and 86 sub-genotypes worldwide^3,4^. The RNA-dependent RNA polymerase (RdRp) is a highly error-prone enzyme as it has no proofreading mechanism, resulting in a high sequence diversity in the newly replicated HCV genomes^5^. Genotypes 1, 2, and 3 are globally distributed, causing 90% of HCV-associated liver infections, while the rest of the genotypes are only found in specific regions. It is pertinent to note that globally distributed genotypes are unevenly prevalent in different regions. Genotype 1 is more prevalent in the United States, Europe, Japan, and Australia. In contrast, genotype 2 is the most prevalent in South America, China, and Japan, and Genotype 3 is common in India, Pakistan, and Afghanistan^6,7^.

Transmission of HCV occurs when blood or other body fluids from an infected person are introduced into the body of an uninfected person. This can occur through sharing syringes in drug abuse, needle-stick injuries or transmission from an infected mother to child during childbirth. Sexual transmission can also occur but is much less efficient than transmission through blood contact^8^. Hepatitis C is not transmitted through kissing, hugging, breastfeeding, sharing utensils or drinking glasses, coughing, sneezing, or casual contact^9^.

Most HCV infections are usually asymptomatic, however, 20% of the infected patients manifest symptoms, such as jaundice, fatigue, and upper abdominal pain. About 80% of the infected patients remain asymptomatic which leads to chronic conditions after many years of the primary infection, and develops the liver cirrhosis, and hepatocellular carcinoma^10^. To date, there is no vaccine available against HCV. Standard HCV transmission barrier precautions includes use of gloves, avoiding close household contact with infected persons, protected sex, and avoiding drug abuse can reduce the possibilities of infection^11^.

Various steps of the viral life cycle are potential targets for drug development against HCV. Since the early 2000s, pegylated interferon and ribavirin have been used to treat HCV, but efficacy was not encouraging due to low sustained virologic response (SVR), and led to severe side effects, such as hemocytopenia, eruptions, and psychotic disorders^12,13^. A deeper understanding of HCV enabled the development of Direct-Acting Antiviral Drugs (DAAs) targeting non-structural proteins (NS5A, NS5B, and NS3) involved in crucial steps of the HCV life cycle^14^. DAAs approved so far include NS5A inhibitor, Daclatasvir, Ledipasvir, and Omitasvir, and a NS5B nucleotide inhibitor (NI) Sofosbuvir. In addition, the non-nucleotide inhibitors (NNI), Dasabuvir, NS3 protease inhibitors, Boceprevir, Telaprevir, Paritaprevir, and Simeprevir, have also been approved for HCV therapy^15–17^. However, due to higher mutation rate in HCV, certain amino acid variations have resulted in resistance against the existing drugs^5^. These resistance-associated mutations can escape the selection pressure during the DAAs therapy, causing a reduced clinical response, and treatment failure^18–20^. Therefore, it is necessary to identify geographic differences with respect to the prevalence of drug resistance sub-genotypes of HCV to achieve the most effective treatment options for patients.

Various studies have been carried out to measure the frequency of resistance-associated mutations in NS3, NS5A, and NS5B of HCV genotypes 1a, and 1b. However, not much data is available to date regarding the genotype 3a, which makes the current study important^21–25^. In Pakistan, mainly Sofosbuvir (Sof) and Daclatasvir are being used for the treatment of HCV-infected individuals^26^. Still, resistance-associated mutations to DAA have not been characterized, to date.

The objective of the present study was to identify the resistance-associated mutations to DAA in the NS3, and NS5B regions in chronically infected patients with HCV genotype 3a in Sindh, Pakistan. This data would help to improve the selection of adequate DAA regimens in future programs to control, and eliminate HCV in the population of Pakistan.

## Material and methods

### Ethical approval and patient’s recruitment

This study was approved by the Institutional Ethical Committee (IEC) of the International Center for Chemical and Biological Sciences (ICCBS), University of Karachi (Study #: -059-HB-2021, Protocol #: ICCBS/IEC- 059-HB-2021/Protocol/3.0, Version #: 3.0). This study was conducted in accordance with the principles of the Declaration of Helsinki. A written informed consent was obtained from each patient prior to the collection of blood sample. A questionnaire was also filled in to obtain the required information. A total of 233 patients, chronically infected with HCV, were recruited at different hospitals in Karachi, Sindh, Pakistan for inclusion in this study. Only patients naive for DAAs treatment were included in the study. Patients co-infected with hepatitis B virus (HBV) and/or human immunodeficiency virus (HIV) were excluded from the study. Patients whose treatment was started before study were also excluded from this study. About 5 mL peripheral blood samples from the recruited patients were collected in yellow-topped blood collection tubes.

### RNA extraction and cDNA synthesis and amplification

HCV RNA was extracted from the serum samples by using QIAamp Viral RNA Mini Kit (Qiagen, Germany), according to the manufacturer's protocol with slight modifications. The concentration of isolated RNA was estimated using high sensitivity RNA kit by qubit fluorometer (Invitrogen, USA). The extracted RNA was stored at – 80 °C until processed for cDNA synthesis. Single-stranded cDNA was synthesized by random hexamers using RevertAid First Strand cDNA Synthesis Kit (ThermoFisher Scientific, USA). Concentration of the synthesized cDNA was evaluated by using a Qubit dsDNA High sensitivity kit.

The HCV NS3 region was amplified by nested PCR using outer and inner primer sets (Table 4)^27^. Initially the synthesized cDNA was amplified by outer primers using an absolute master mix containing; Taq polymerase, dNTPs mix, buffer, etc. (Molequlon, New Zealand), and the PCR conditions were set as follows; initial denaturation at 95 °C for five minutes, followed by 35 cycles of 95 °C for 30 secs, 52 °C for 25 secs, and 72 °C for one minute, and then final extension at 72 °C for five minutes. For second round of amplification, the inner primer annealing temperature was set at 55 °C for 40 s. HCV-NS5B region was amplified using the set of primers enlisted in Table 1^28^. PCR was performed using the following conditions; denaturation at 95 °C for 5 min, followed by 35 cycles of 95 °C for 25 s, 55 °C for 25 s, and 72 °C for 60 s, and final extension at 72 °C for 5 min, and the reaction was held at 4 °C. The amplified products were evaluated by using 2% agarose gel electrophoresis.

**Table 1:** Primers for amplification of NS3, and NS5B regions in genotype 3a.

Primer	Sequences	Polarity
Primers for amplification of NS3 region in genotype 3a		
F1	5′-GTCTCTGCRCGATTAGGCCGTGA-3′	Sense strand
R1	5′-CAGTTTRGCACCAGTTGTAACG-3′,	Antisense Strand
F2	5′-GTTGGGACCTGCTGATGACTA-3′	Sense strand
R2	5′-CCCAGTGCGGATGTTGGGGT-3′	Antisense strand
Primers for amplification of NS5B region in genotype 3a		
F1	5′-GTSTGGIARGACYTICTGGAAGAC-3′	Sense strand
R1	5′-GTSTGGIARGACYTICTGGAAGAC-3′	Anti-sense strand

The PCR products were purified by using Agencourt Ampure XP beads (Beckman coulter, USA). For purification, 1.8 volumes of magnetic beads were mixed with the PCR products, and then incubated for 5 min at room temperature. The tubes were placed in the magnetic rack, and the supernatant was discarded. Beads were washed two times with 70% ethanol, followed by DNA elution.

### Sanger sequencing

The purified DNA was subjected to Sanger sequencing using an inner set of primers, and both reverse and forward primers were used to generate consensus sequences. The sequencing reaction was performed by BigDye™ Terminator v3.1 Cycle Sequencing Kit (Applied Biosystems, USA), according to the manufacturer’s protocol. The sequencing products were purified by using the ethanol precipitation method. The purified products were sequenced by using a 3500 genetic analyzer (ThermoFisher Scientific, USA).

### Data analysis

The sequencing data of NS3, and NS5B genes were analyzed using BioEdit 7.2 software^29^. The sequencing data were compared with HCV genotype 3a reference genome (Accession ID = D17763). The obtained sequences were analyzed by using Geno2Pheno software to find out resistance-associated mutations^30^. The virus pathogen resource (ViPR) database was used to recover the sequences of NS3, and NS5B of HCV genotype 3a^31^. The following information was obtained for each sequence (accession number, times of sampling and publication, human host, HCV genotype/subtype, geographical location). We downloaded 1696 sequences of NS5B, and 1100 of NS3 regions of HCV3a from the ViPR database. Low-quality data and duplicated sequences were removed. The filtered sequences were analyzed to identify the prevalence of resistance associated mutation (RAS) in various geographic regions. The identified resistance-associated mutations were further analyzed for the occurrence among predominant HCV genotypes; 1a, 1b, and 2a. Multiple sequence alignment of 35 sequences from the current study, and 800 sequences across the global population including 335 from Asia, 311 from Europe, 84 from North America, and 70 sequences from Oceania of NS5B HCV 3a was performed using muscle alignment tool^32^. The phylogenetic tree was constructed by RAxML 8.2.12 tool by using the maximum likelihood (ML) method and 1000 bootstrap replicates^33^. The phylogeny tree was further elaborated with FigTree v.1.4.4^34^ and MEGA X software^35^.

### Statistical analysis

For continuous data, results are shown as median values and interquartile range (IQR), while for categorical data, results are presented as a number (%). When applicable, the Chi-squared test was used to compare categorical variables. Unpaired T-test was performed in the group wise comparison (male *v.s.* female; younger age *v.s.* older age). Statistical analysis of clinical data was performed using the R program, RStudio Team (2020). RStudio: Integrated Development for R. RStudio, PBC, Boston.

### Ethical approval

This study was designed according to the Helsinki guidelines, and approved by the Institutional Ethical Committee (IEC) of the International Center for Chemical and Biological Sciences (ICCBS), University of Karachi (Study #: -059-HB-2021, Protocol #: ICCBS/IEC- 059-HB-2021/Protocol/3.0, Version #: 3.0).

## Results

### Characteristics of the study population

The study cohort comprised 233 patients (male = 110, female = 123), chronically infected with HCV. The age of the study participants ranged between 11 and 80 years (average = 44.71 ± 14.28, median = 45 years). The average age in the female patients was 45.61 ± 14.42 years, and the median age was 45 years. Likewise, in the male patients, the average age was 43.69 ± 14.19 years, and the median age was 45 years (Fig. S1). The clinical, and virological data are summarized in Table 2.

**Table 2:** Demographics, and baseline clinical characteristics.

Clinical and virological data	Range
Age in years (mean)	45 (11–80) ± 11.05
Male	110
Female	123
HCV genotype	3a
ALT	52 ± 6.534
AST	43 ± 4.05
No information	7/51 (13.7)
HCV viral load mean	Log 6.7 ± 3.5

### Analysis of the viral load

**Figure 1:**
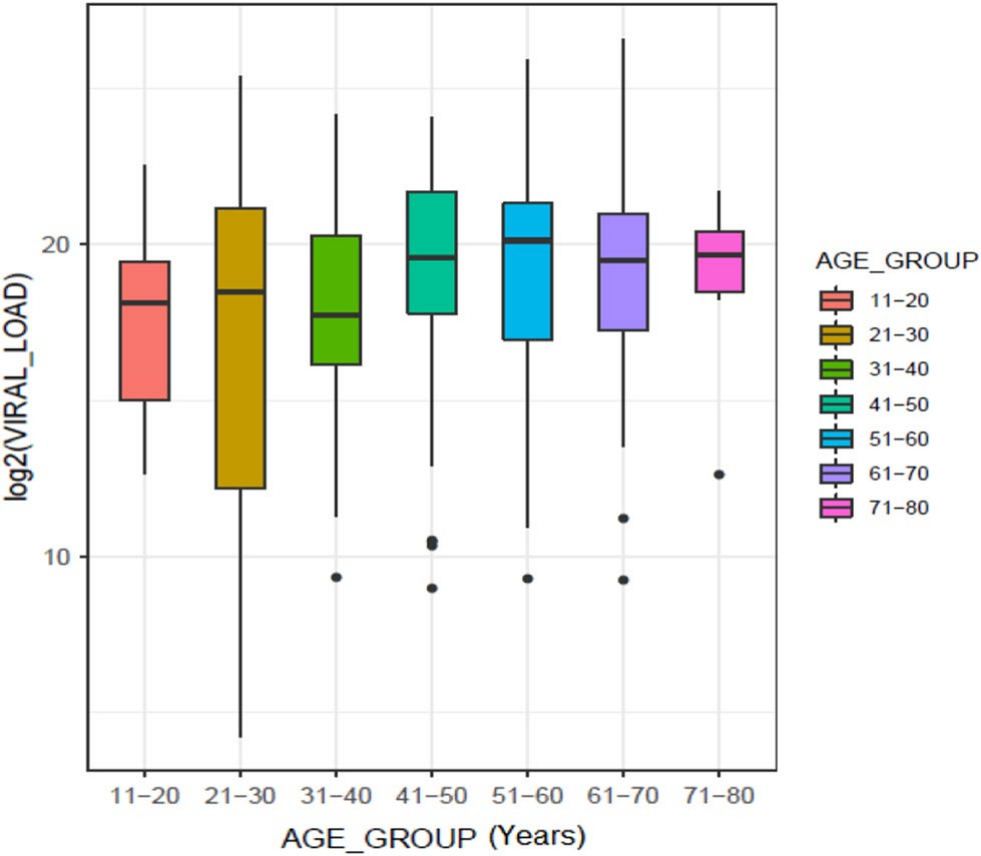
Viral load in patients of different aged groups. The median viral load was slightly higher in the elderly patients than in the younger patients (age ≤ 40 years).

The viral load in each sample was determined through real-time PCR, and the Ct value was used to calculate the viral load (IU/mL) by using a standard curve. The viral load ranged between 1.9 _×_ 10^1^ – 1.002 _×_ 10^8^ U/mL, with an average viral load of 3.4 _×_ 10^6^ U/mL, and a median of 4.96 _×_ 10^5^ U/mL. Analysis of the viral load in the male, and female patients indicated a similar load (P = 0.33, Fig. S2). There was a non-significant exponential correlation between the age of patients and the viral load, R^2^ = 0.0239 (Fig. S3). Analysis of the viral load across different age groups (11–20, 21–30, 31–40, 41–50, 51–60, 61–70, and 71–80 years) revealed a slightly higher viral load in the mean aged patients (Fig. 1), which was might be due to the compromised immunity in elder ages.

### Analysis of resistance associated mutations (RAM) and genetic similarity across different geographic regions of the world

We were able to amplify 50 out of the 233 samples by using the specific set of primers of NS3, and NS5B regions. The amplicons were Sanger sequenced for the identification of RAM (Resistance associated mutations) in Pakistani patients. Despite multiple attempts, the remaining samples (183) could not be amplified due to either the lower viral load or higher degradation of the viral RNA.

**Figure 2:**
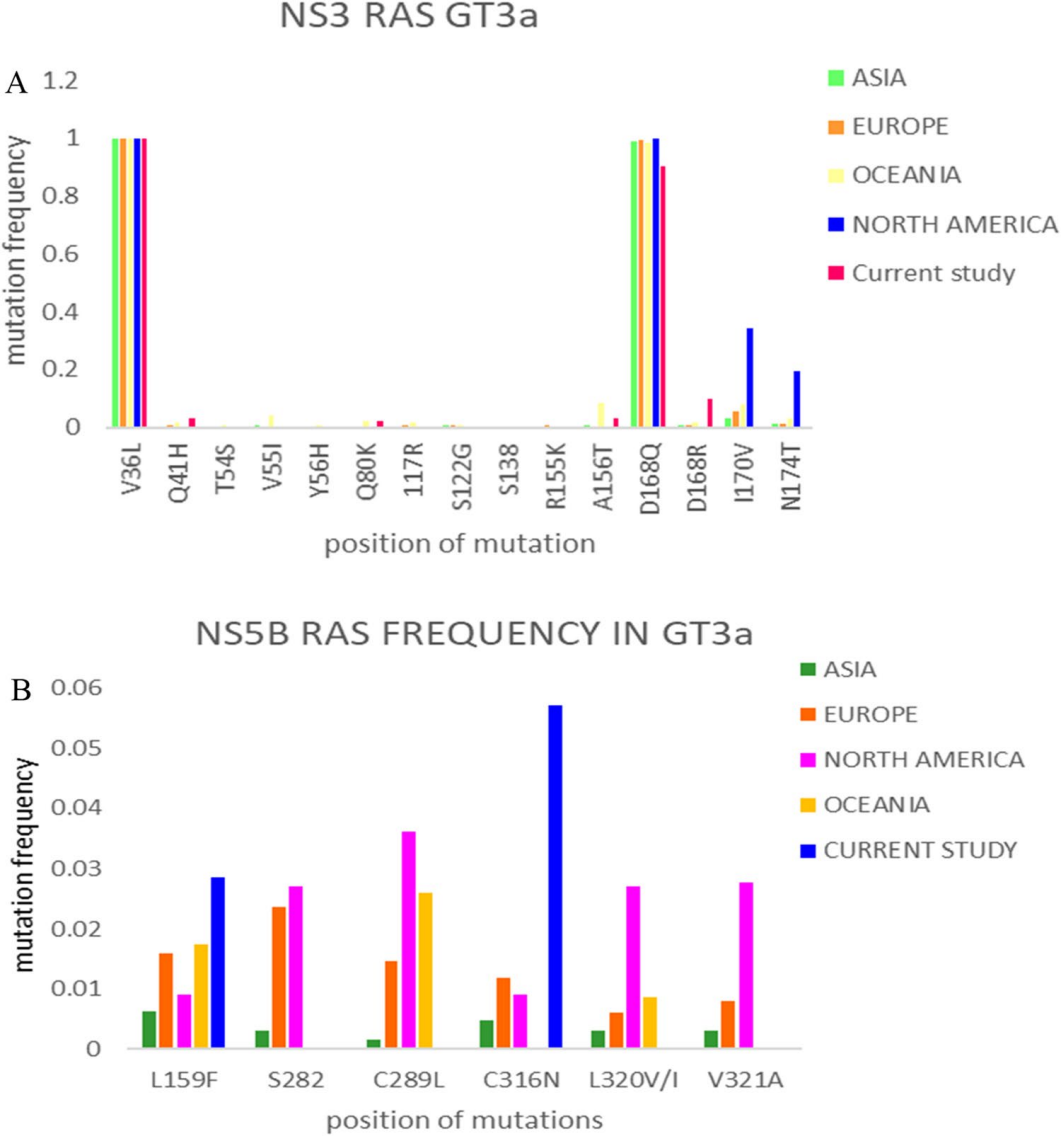
(**A,B**) Comparison of the mutation frequencies in the genotype 3a across different geographical regions.

NS3 RAM were analyzed in 31 HCV-infected patients, while NS5B RAM was analyzed in 35 patients, according to the inclusion and exclusion criteria of the study. Overall, fifteen NS3 positions were analyzed for RAM detection, and on three positions (Gln80, Ala156 and Gln168) amino acid substitutions were observed. Resistance-associated mutations were identified on amino acid positions including Ala156Tyr, Gln80Glu, and Gln168Arg. The overall prevalence of these mutations was found 22.5%. The Val36Leu mutation was found in all the patients, whereas, the Asp168Gln mutation was present in 90% of the patients. In our study population, RAM including Gln168Arg was present in 9.6%, Glutamine 80 (Lys and Glu) was found in 6.4%, while two mutations Gln41His, and Ala156Thr were detected in 3.2% in the sequenced samples. Multiple mutations in NS3 region were not found in any sequenced sample. We compared the in-house sequenced data with the publicly available HCV sequenced genomes. A number of 1593 HCV 3a NS3 region sequences, including in house sequenced samples (n = 31), Asian (n = 332), European (n = 356), North American (n = 748), and Oceania (n = 128) were analyzed for RAM, and the data is shown in Fig. 2, and Table 2. Two amino acid substitutions including Val36Leu, and Asp168Gln were present in all populations (prevalence 100%). These are genetic resistance markers of genotype 3a. The rest of the mutations were present with a low prevalence. Substitutions in Thr54, and Tyr56 associated with resistance to first-generation protease inhibitors were rare (2/1593, 3/1593, respectively) in all geographic regions. The Val55 to Ile/Ala substitutions are associated with resistance; and were also present in low prevalence (8/1593) among the analyzed sequences. The Gln80Lys is associated with resistance against Simeprevir and was present in 2.8% in Oceania, but rare in other geographic regions (1/332 Asia, 1/356 Europe). The Arg117His was not present in any population. The Ser122Thr was also present in low prevalence. Ser138 was conserved in all geographic regions. The Arg155 was reported only in European samples (2/356). The Ala156Thr, associated with Glecaprevir, and Voxilaprevir resistance was present in (1/31) of in-house data, and Oceania (2/126).

We found Gln at position 168 to Arg, which is associated with resistance to Glecaprevir, in three patients of the studied subjects, and in two of the 256 sequences of the European continental regions. The mutation valine to isoleucine at position 170 was prevalent with various frequencies, while resistant associated mutations including Ala/Tyr/Gly/Leu/Met were not found in NS3 HCV genotype 3a. Similarly, at position 174, three mutations (Ser/Ala/Iso) were present in the available sequenced data while Phe/Pro resistance mutations were absent. The resistance associated mutations have significant role in reducing the virus susceptibility against direct acting antivirals. The prevalence of identified mutations is shown in Table 3.

**Table 3:** Prevalence of NS3 across different geographic regions.

Position	Pakistan	Asia	Europe	North America	OCEANIA
Val36Leu	30/31	332/332	356/356	748/748L	125/128Leu 3/128Ala
Gln41Arg	1/31	1/332	3/355	–	–
Thr54Ser	31 T	–	1/355	–	1/128
Val55Ile	–	2/332	1/355	–	1/128
Tyr56Phe	–	–	1/355	–	1/128
Gln80Lys/Glu	2/30	–	1/355	–	3/128
Arg117His	–	–	356Arg	–	2/128
Ser122Thr	–	2/332	1/355	–	1/128
Arg155Thr	–	–	2/355	–	–
Ala156Thr	1/31	2/332	1/355	2/748	–
Gln168Arg	3/31	1/332	2/355	2/748	2/128
Ile170Thr	–	–	–	–	10/128
Thr174Ala	–	328Thr 3Ala/1Ser	5/355	146/748	4/128

For NS5B RAM, five important positions, including 159, 282, 289, 316, and 320 were analyzed. The amino acid substitutions were detected in positions 159, and 316. The resistance associated mutation Cys to Asp at position 316 was observed in 5.7% samples of the current study sequences. Furthermore, the resistance associated mutation Leu159 to Phe was observed in 2.8% samples of the current study sequences. We did not observe any resistance associated mutation at positions 282, 289, and 320 in the analyzed samples.

In comparison to Asia, Europe, North America, and Oceania, Pakistan has nearly the same prevalence of RAS's NS5B mutations (Fig. 2, Table 4). The overall prevalence of Sofosbuvir-based RAS is low in genotype 3a patients in treatment naïve patients^36^. Pakistan had a higher prevalence of Cysteine to Asparagine at position 316 than other populations. However, because of the large genetic barrier to resistance, this does not confer Sofosbuvir resistance^37^. RAM is more common in Sofosbuvir treatment failure patients. RAM analysis for other genotypes was also carried out across various genotypes (1a,1b) (Figs. S4–S7).

**Table 4:** Prevalence of NS5B RAS across different geographical regions.

Position	Pakistan	Asia	Europe	North America	Australia
Leu159Phe	1/35	4/302	2/125	1/81	2/85
Ser282Thr	–	2/628	8/339	3/81	–
Cys/Phe/Met 289 Ile/Leu	–	1/628	5/339	4/81	3/85
Cys316Arg	2/35	3/628	4/339	1/81	–
Leu320 Phe/Ile/Val	–	2/628	–	3/81	1/85
Val321Ala/Ile	–	2/628	–	3/81	–

### Phylogenetic analysis

**Figure 3:**
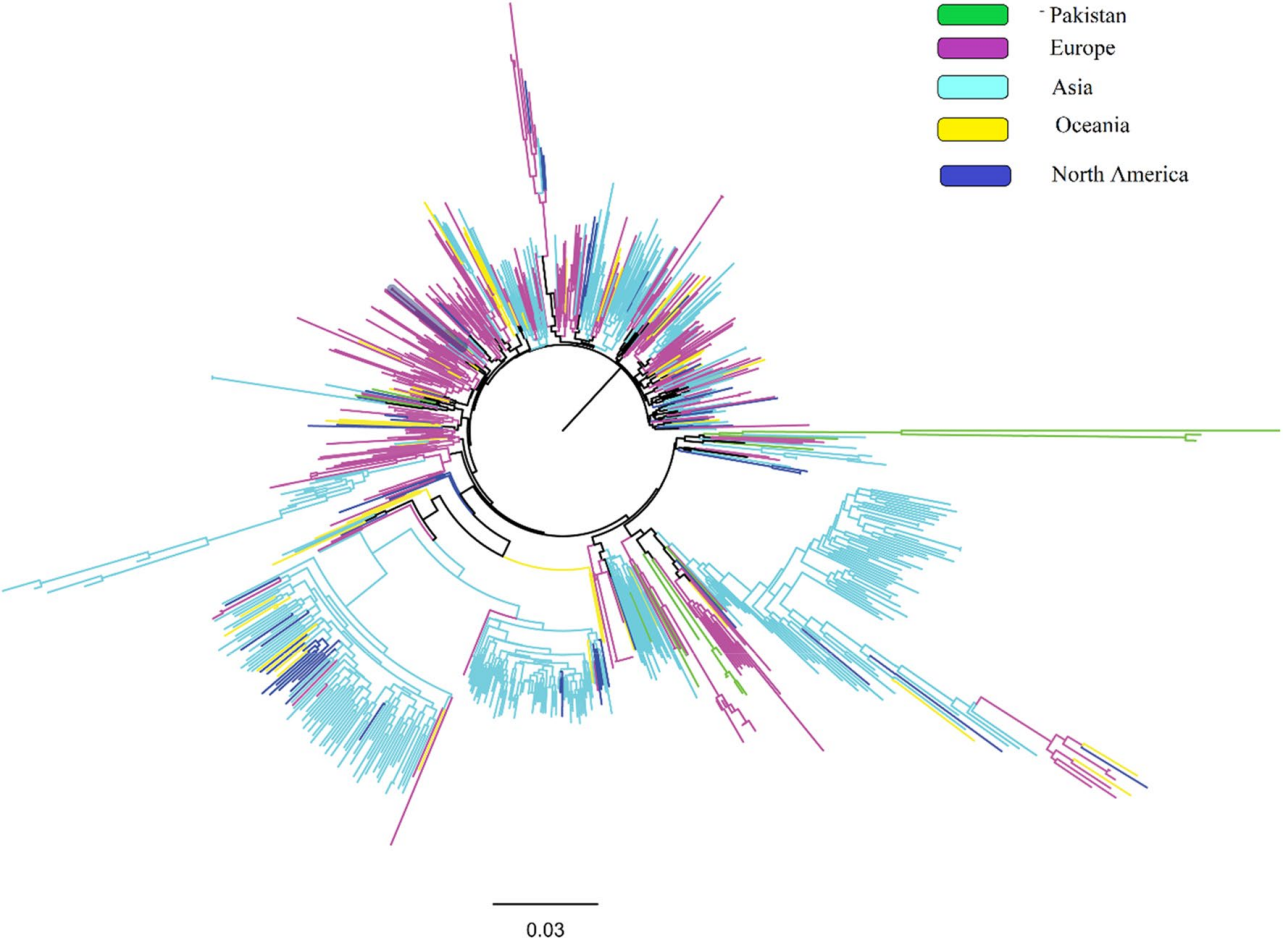
Phylogenetic analysis of HCV in Pakistan to characterize genetic similarity to other countries. Maximum likelihood phylogenetic trees were inferred for genotypes GT3a. Tips are color-coded, with Pakistan in green, European countries in pink, countries North America in blue, countries in Asia in shades of green and Oceania in purple). For simplicity of visualization, countries are grouped together as part of that geographical region. Phylogenetic trees of HCV sequences from different regions including Pakistan are shown.

The NS5B genotype 3a phylogenetic tree included 835 sequences (in house = 36, Oceania = 70, North America = 84, Asia = 345, Europe = 300). Same sequences were used for RAM analysis in NS5B (Sequence length was 400 bp (8208–8596) D17763)). NS5B Genotype 3a sequences from Pakistan formed 3 clusters. One cluster was monophyletic and distant, and the other contained sequences from Asia and Europe. Overall, a cluster of our sequences was more diverse in our sequences (Fig. 3).

The phylogenetic tree revealed a high genetic diversity among all sequenced strains. HCV sequenced from the Asian population exhibited high genetic diversity, and mostly grouped in the separated clades along with few other strains of mostly European strains. Five in house sequenced strains showed high genetic diversity, and forming a new sub-branch, and placed with other strains, mostly isolated from Europe, and Asia.

## Discussion

The prevalence of drug resistance associated mutations varies between different HCV genotypes^25^. There is no established standard test for identifying resistance associated mutations. There is also no consensus on such testing, and it seems difficult to implement such type of testing^38^. The current study presents data on NS3, and NS5B RAS genotype 3a in Pakistan. Various studies have been carried out to identify resistance associated mutations in genotypes 1a and 1b, but data is limited regarding genotype 3a, as the most common genotype prevalent in Pakistan. In the present study, overall prevalence of NS3 resistance associated mutations is 22.5% for genotype 3a in Pakistan. A recent study conducted on HCV genotype 3a from the northern region of Pakistani population reported that the prevalence of resistant mutation was found to be 30% in the NS3 region^39^. Two mutations including Valine at position 36 to Leucine and Aspartate at position 168 to Glutamine detected in all studied NS3 sequences. These mutations are considered as genetic resistance markers unique to genotype 3a, and these increase resistances to the first-generation protease inhibitors (Boceprevir, and Telaprevir) up to 700-fold. Due to these natural mutations, these drugs are directed primarily against genotype 1^40,41^. Mutations in the NS3 genotype 3a at positions Thr54, and Tyr56 were not found among the studied sequences while found in the few samples of the publicly available database. Earlier study reported that Thr54Ser was found in the genotype 1a in Iranian patients^42^. The prevalence of Gln80Lys natural polymorphism, related to resistance to Simeprevir, is high in genotype 1a patients was also observed in the current study^43,44^. The prevalence of Ala156Thr was 3.2%, associated with resistance to pan genotypic protease inhibitors Glecaprevir. However, this variant displayed poor replicative fitness and diminished following drug selective pressure^45^. The frequency of Glutamine at 168 to Arginine polymorphism was as high as 9.6%, which is associated with resistance to Simeprevir, and Glecaprevir^46^. Asp168Gln was present in all patients in other studies, and any different mutation was rarely found^40^. In this study, Val170Ile substitution was present in the patients, similar to the previous studies. However, this substitution is not associated with drug resistance^40,47^.

In this study the observed frequency of NS5B RAS was 8.5% in genotype 3a. Gaudieri, S., et al. in 2009 reported that 10% resistance associated substitutions in the NS5B region^48^. In HCV 3a, Serine at position 282 to Threonine/Alanine in the NS5B was not detected in the clinical studies or any other real-world cohort studies. This mutation was only reported in *in-vitro* studies^49^. Leu at position 159 to Phe was found in 2.8% of patients however did not affect the efficacy of Sofosbuvir treatment^50,51^.

In the present study, Cys316Asn was present in 6% of patients. This substitution in along with Leu159Phe was detected in GT1b and GT3 in the patients who failed Sofosbuvir treatment^51,52^. These mutations have very low fold changes for Sofosbuvir and Dasabuvir^50,53^. The impact of these mutations is not yet fully understood, but they have been reported to occur more frequently in patients who have experienced treatment failure^45,54–56^. The phylogenetic analysis suggests that genotype 3a was originated from Asia. In accordance with data as genotype 3a is endemic in these regions, and genotype 3a in injectable drug users in Europe was observed as similar to Asia^57,58^. The current study was limited to only one region of Pakistan and only treatment naïve patients and chronically infected with HCV were included in this study. Future studies should be focused on the natural prevalence of RAMs in Pakistan, as well as analysis of RAMs produced during the HCV treatment. The present study may be helpful in determining the best therapeutic strategy with DAAs for HCV patients.

## Conclusions

The present study indicates that resistance-associated substitution is not frequent in NS5B; however, mutations in the NS3 region are present in considerable numbers. No resistance was observed against Voxilaprevir, a pan-genotypic protease inhibitor, which indicates that could be a highly effective therapy in this region. The practical implementation of these findings poses challenges due to the limited availability of genotypic resistance testing and the constraints imposed by costs and reimbursement policies on DAA prescriptions. However, in countries with a high prevalence of HCV infection and access to validated RAS testing, understanding the natural burden of RASs in specific HCV genotypes and subtypes can guide targeted HCV diagnostic interventions in clinical conditions and settings. This approach aims to optimize the efficacy of DAAs, potentially reducing the use of incorrect and costly therapies.

## Supplementary Information

Supplementary Figures.

## Data Availability

The datasets generated and/or analyzed during the current study are available in the NCBI repository, with the accession numbers ON603922 to ON603949.

## Abbreviations

HCV: Hepatitis C virus
RAS: Resistance associated substitution
DAA: Direct acting antiviral
NS: Non-structural
RdRp: RNA-dependent RNA polymerase
SVR: Sustained virological response
