# Supplementary material for: Study of drug resistance-associated genetic mutations, and phylo-genetic analysis of HCV in the Province of Sindh, Pakistan
Source: Sci Rep. 2023 Jul 27;13:12213. doi: 10.1038/s41598-023-39339-4 (PMC10374889; doi:10.1038/s41598-023-39339-4)
Supplement: Supplementary file 1 — Supplementary Figures. [file 41598_2023_39339_MOESM1_ESM.pdf]

Supplementary Figures

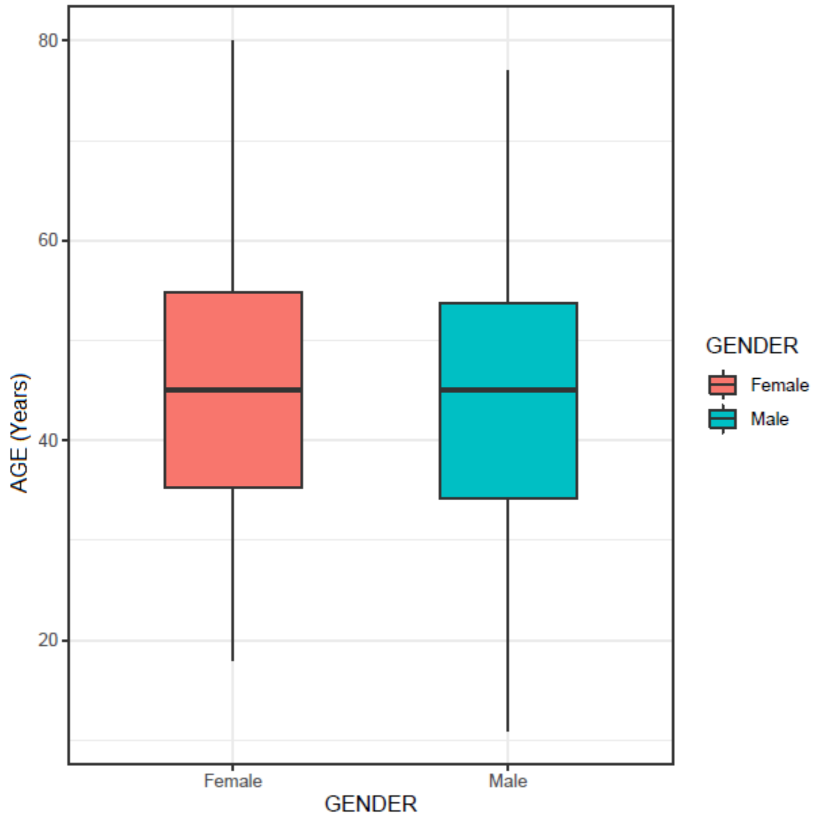

Supplementary Figure S1: Distribution of the ages of the patients of this study.

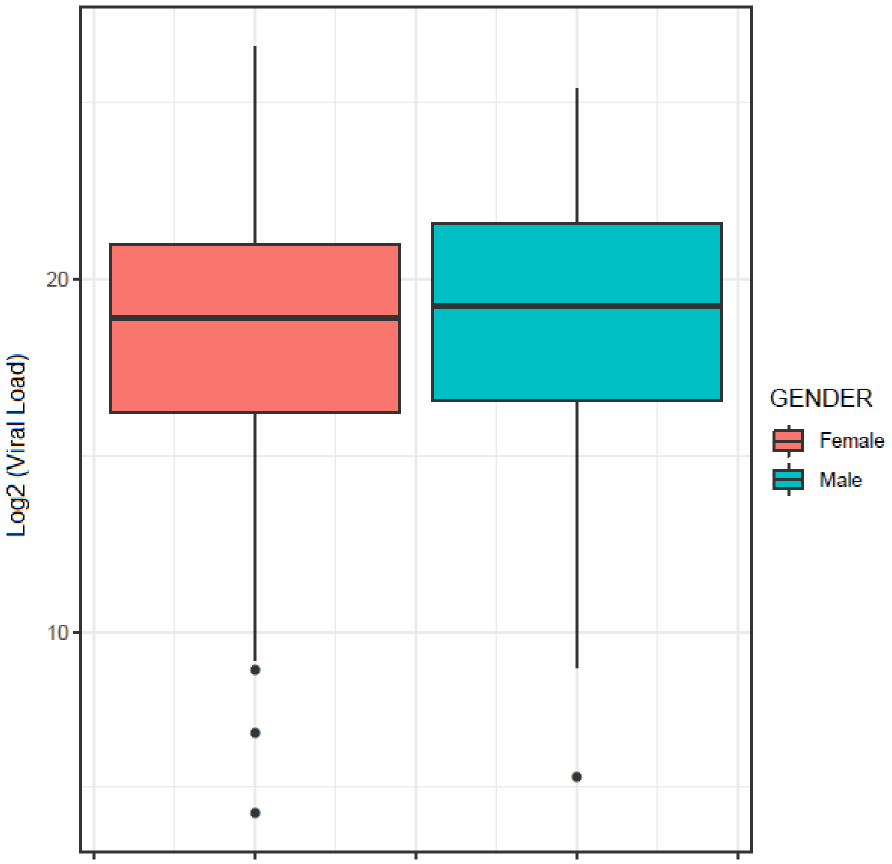

Supplementary Figure S2: Distribution of the viral load in the male and female patients.

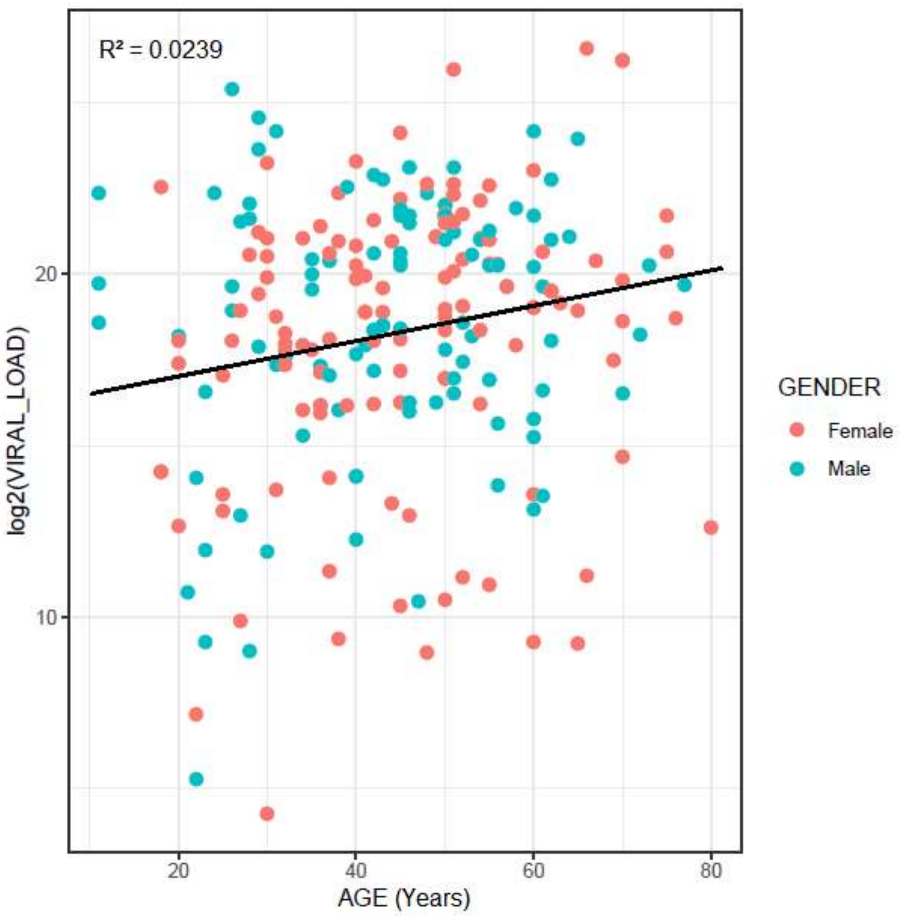

Supplementary Figure S3: Correlation of the age of the patients with the viral load. There was a non-significant correlation between these two variables.

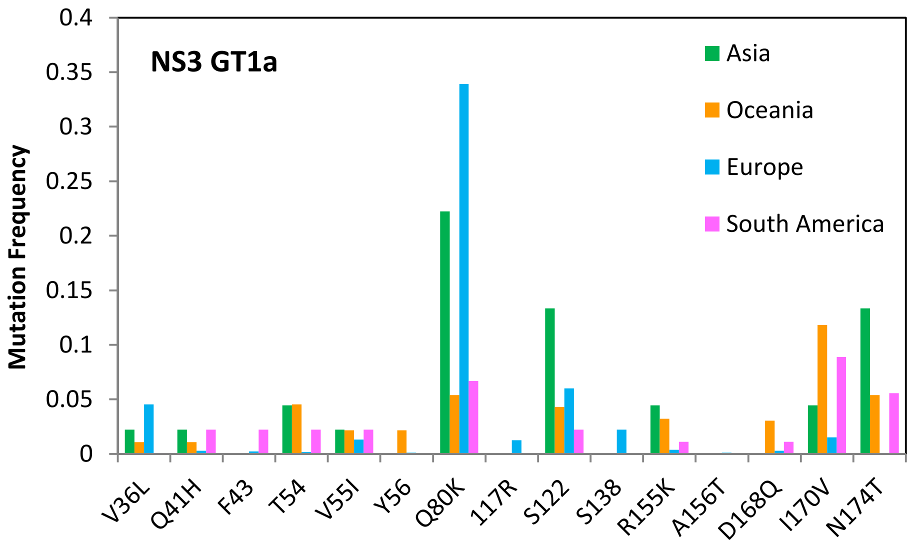

Supplementary Figure S4: Comparison of NS3 resistance associated mutations in genotype 1a across different geographic regions

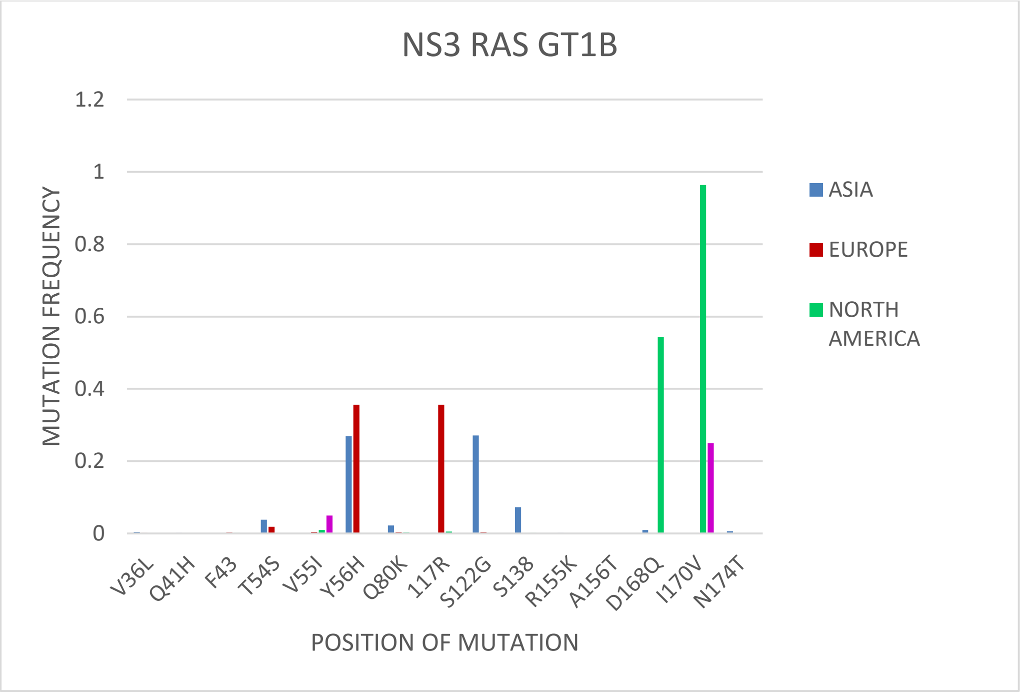

Supplementary Figure S5: Comparison of NS3 resistance associated mutations in genotype 1b across different geographic regions

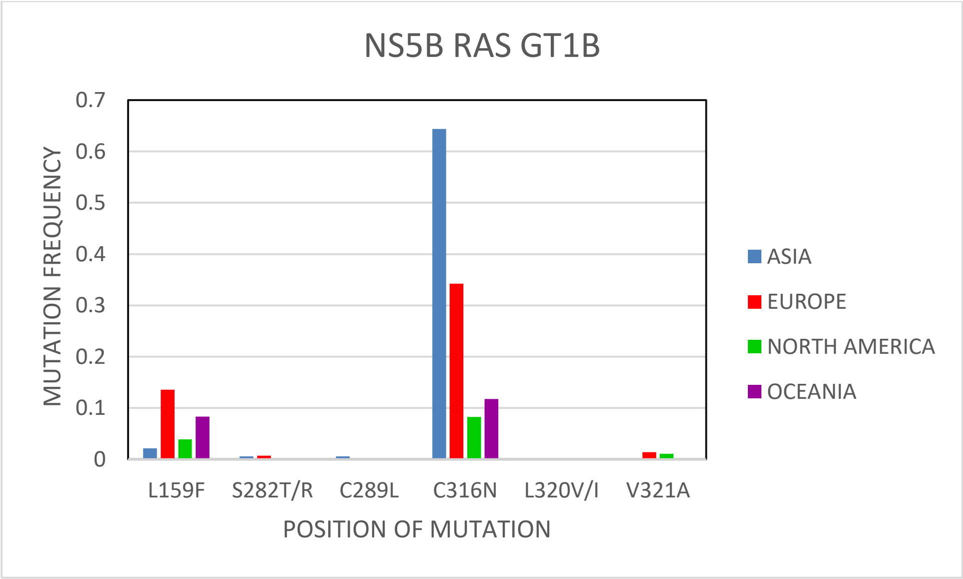

Supplementary Figure S6: Comparison of NS5B resistance associated mutations in genotype 1b across different geographic regions

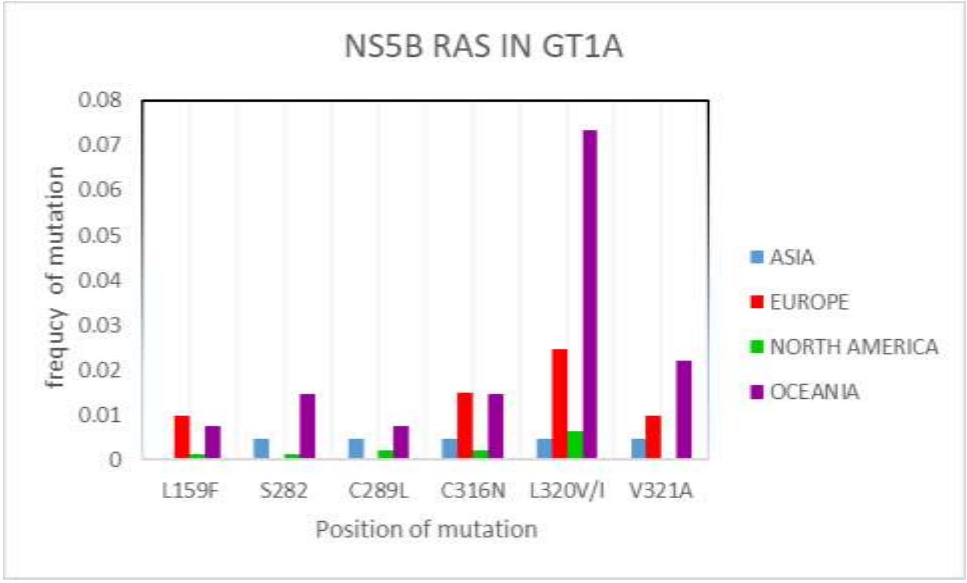

Supplementary Figure S7: Comparison of NS5B resistance associated mutations in genotype 1a across different geographic regions
